# Insight into the Mechanical Properties and Microstructure of Recycled Aggregate Concrete Containing Carbon Fibers and Nano-SiO_2_

**DOI:** 10.3390/ma17225633

**Published:** 2024-11-18

**Authors:** Tong Xing, Shaofeng Zhang, Lei Guan

**Affiliations:** 1 School of Architecture Engineering, Jilin Engineering Vocational College, Siping 136001, China; xingtong192@163.com; 2 Shaanxi Railway Institute, Weinan 714000, China; 3 China Construction Third Engineering Bureau Group Co., Ltd., Nanjing 210046, China; snkeg1987@163.com

**Keywords:** carbon fibers, nano-SiO_2_, recycled aggregate concrete, mechanical properties, microscopic characteristics

## Abstract

This study aimed to improve the mechanical properties and microstructure of recycled aggregate concrete (RAC) by incorporating carbon fibers (CFs) and nano-SiO_2_ (NS) to promote the optimal utilization of RAC. The mechanical properties of the RAC were enhanced by both single and hybrid additions of CFs and NS, and the hybrid addition had a better strengthening effect. From the experimental results, it was found that the addition of CFs could increase the 28 d compressive strength and splitting strength of the RAC by 9.05% and 22.36%, respectively. The hybrid CFs and NS were more conducive to improving the mechanical properties of the RAC, and the enhancement effect increased first and then decreased with an increase in the NS content. The optimal content of NS was 0.8 wt%, which increased the 28 d compressive strength and splitting strength of the RAC by 20.51% and 14.53%, respectively. The microstructure results indicated that the addition of CFs had little effect on the optimized pore structure of the RAC, but the crack inhibition action of the CFs could improve the mechanical properties of the RAC. The addition of NS reduced the content of CH and facilitated the formation of more (C–S–H) gel. The hydrated calcium silicate (C–S–H) gel significantly decreased the porosity and transformed harmful capillary pores and harmful pores into harmless capillary pores and gel pores, thus improving the mechanical properties of the RAC. Therefore, the use of hybrid CFs and NS was more conducive to enhancing the performance of RAC for building materials.

## 1. Introduction

With the rapid development of urbanization, construction and demolition (C&D) waste has increased rapidly in recent years [1,2]. According to statistics, the C&D waste in China amounted to about 3.5 billion tons in 2021 [3]. However, technology for the utilization of C&D waste is relatively poor, and the utilization rate of C&D waste is ~5–10%, resulting in serious environmental problems. The reuse of C&D wastes to prepare recycled coarse aggregate concrete (RAC) could not only effectively solve the problems of construction resources, energy, and the environment, but also contribute to environmental and resource preservation, as well as realize the sustainable development of the construction industry [4,5]. However, RAC particles have a significantly different surface structure compared with that of natural coarse aggregate (NCA) particles due to the existence of adhered mortar, leading to the inferior performance of RAC in terms of its mechanical properties, microscopic characteristics, durability, etc. [6,7]. In order to improve the practical application efficiency of RAC, enhancing the strength and compactness of RAC has been attracting considerable attention for a long time.

At present, the addition of fibers to reinforce materials is internationally considered to be an effective measure with which to improve the mechanical properties of RAC, increasing the toughness and crack resistance of RAC by bridging cracks and alleviating the rapid generation and development of microcracks in the matrix [8,9]. CFs are a kind of lightweight and high-strength fiber material that have a typical density of 1.6–2.5 g/cm^3^, a tensile strength of 2.2 GPa, and an elastic modulus of 200–350 GPa [10]. Moreover, CFs are temperature-stable and corrosion-resistant. In addition, the active hydroxyl and carboxyl groups on the surface of CFs could provide strong adsorption of the hydroxyl groups in cementitious materials, which would increase the bonding performance between CFs and cementitious materials [11,12]. Therefore, the appropriate amount of CFs could improve the tensile properties, flexural properties, and toughness of cementitious materials, while enhancing their flexural impact resistance. Guo et al. [13] reported that CFs could effectively improve the flexural and split tensile strength of concrete, while their effect on compressive strength was limited. The improvement effect of CFs was optimal when the length of the CFs was 10 mm and the cement admixture amount was 1 wt%. Wang et al. [14] investigated the effect of CF dispersion on the mechanical properties of CF-reinforced concrete and found that uniformly dispersed CFs with a content of 0.6 wt% increased the compressive strength by 10% and the modulus of elasticity by 26.8%. Wang et al. [15] investigated the effects of CFs on the mechanical properties of RAC and found that CFs at a content of 0.35 wt% and a length of 6 mm significantly increased the tensile strength, but the enhancement effect on the compressive strength was not obvious. Yan et al. [16] found that the CFs not only played a role in carrying the load through the bridging and making the crack extension paths more complex, but also refined the pore structure of the RAC via their crack-blocking effect. Therefore, it can be concluded that the addition of an appropriate quantity of CFs is beneficial to enhance the toughness, resistance to cracks, and ductility of cementitious materials, but not significant in their compressive improvement.

To improve the mechanical properties and durability deficiencies of cementitious materials, nanomaterials are generally regarded as ideal reinforcing materials for reducing the defects of cement-based materials at the nanoscale [17,18]. Nanomaterials, as a new type of inorganic material with high efficiency, such as nano-SiO_2_ (NS) and nano-CaCO_3_ (NC), are characterized by a small particle size, large specific surface area, strong surface adsorption, and high surface energy [17]. Nanomaterials could accelerate the hydration reaction of cementitious materials, which could significantly optimize the microstructure of cementitious materials through pozzolanic, nucleation, and filling effects. Compared with the other nanomaterials, NS is the most widely used nanomaterial due to the chemical reaction between NS, CH, and C_3_A, which results in the increased formation of hydration products and significantly strengthens cement-based materials [19]. Hu et al. [20] found that the addition of NS facilitated the early hydration reaction of cementitious materials and significantly improved the 28 d compressive strength. Ghafari et al. [21] illustrated that the interfacial transition zone (ITZ) was much denser and the pore structure was more compact as a result of the formation of hydrated calcium silicate (C-S-H) gel via pozzolanic effects, which effectively improved the mechanical properties and durability of concrete. Zaidi et al. [19] discussed the mechanism of the microstructure of RAC modified by NS and found that NS could effectively improve the compactness and permeability of RAC by optimizing the distribution of micro-pore sizes. Ying et al. [22] found that the high pozzolanic activity, filling, and nucleation effects of NS had a substantial impact on the ITZ of RAC and reported that the chloride permeability coefficient of RAC with 2% NS decreased by 25% in comparison to the RAC control group. However, Sivasankaran et al. [23] found that the excessive addition of NS decreased the compactness of concrete due to the agglomeration phenomenon of NS, which could not optimize the microstructure of concrete and led to inferior mechanical properties.

At present, there is more research on the effect of CFs or NS on the mechanical properties of cementitious materials, while the research results on the enhancement of the mechanical properties of cementitious materials by hybrid CFs and NS are relatively limited. Because the diameters of CFs and NS differ by one order of magnitude, hybrid CFs and NS could exhibit a synergistic improvement effect on the properties of cementitious materials at both the nanometer and micrometer scales. Based on previous studies, this paper explored the enhancement effect on RAC of incorporating CFs and NS. Meanwhile, the microscopic characteristics of RAC were investigated via thermogravimetric analysis (TG), X-ray diffraction (XRD), mercury intrusion porosimetry (MIP), and scanning electron microscopy (SEM). Hopefully, this study can provide guidance on the effective utilization of RAC in the construction industry.

## 2. Experimental Procedure

### 2.1. Materials

**Figure 1:**
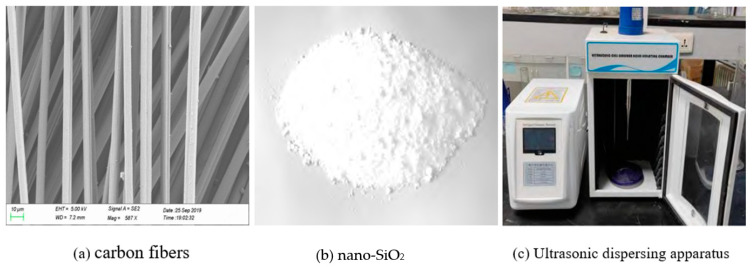
Modified materials and dispersing device.

O 42.5 Portland cement (OPC), provided by Shenyang Jidong Cement Co., Ltd. (Shenyang, China), and fly ash, produced by Shenyang Coal Industry Co., Ltd. (Shenyang, China), were used to make cementitious materials, whose chemical compositions and physical properties are shown in Table 1. The locally available natural river sand was used as a fine aggregate (FA), and its maximum particle size, apparent density, and fineness modulus were 4.75 mm, 2.61 g/cm^3^, and 2.8, respectively. The RAC was produced by Jilin Houde Renewable Resources Co., Ltd. (Dehui, China), and its maximum particle size, bulk density, performance density, water absorption, moisture content, and crushing values were 20 mm, 1568 kg·m^−3^, 2465 kg·m^−3^, 3.05%, 2.0%, and 15.7%, respectively, which were compliant with the Chinese code (DB22/T 5017-2019). CFs had a carbon content of >95 wt%, lengths of 3 mm, diameters of 7 μm, densities of 1.75 g/cm^3^, tensile moduli of 228 GPa, and tensile strengths of 3.53 GPa, and their morphology is shown in Figure 1a. The NS was purchased from McLean Reagent Co., Ltd., and possessed a particle size of (20 ± 5) nm, a specific surface area of 193 m^2^/g, a pH value of 6.9, and a purity of 99.5%, as shown in Figure 1b. NS was dispersed by an HN-500 ultrasonic nanomaterial disperser in the preparation of RAC, as shown in Figure 1c. The dispersion power and dispersion time were 600 W and 15 min, respectively. A polycarboxylic-based superplasticizer (PBS) with a water reducing rate of 30%, solid content of 40%, and alkali content of 6.5% was obtained from Jiangsu Bote New Materials Co., Ltd. (Nanjing, China). The water (W) that conformed to Chinese national standards, JGJ 63-2006, was used for preparing RAC specimens.

### 2.2. Mix Proportions

As reported in previous studies, an appropriate fiber content is the basis for ensuring the maximum performance enhancement of concrete. The authors of [17,20] suggested that 0.6 wt% is the optimal content of CFs. The mechanical properties of concrete were improved with an increasing CF content, as the CF content was less than 0.6 wt% of the cement mass. However, when the CF content was more than 0.6 wt% of the cement mass, the improvement in the mechanical properties of concrete decreased with an increase in the CF content, and the higher the CF content, the more obvious the decrease in the strength enhancement was. This being the case, the content of CFs was chosen to be 0.6 wt% of cement. Meanwhile, previous studies have indicated that the addition of NS could effectively enhance the mechanical properties of concrete, and the content of NS ranged from 0.2% to 1.2%. The primary objective of this paper was to study the influence of CFs and NS on the mechanical properties of RAC at the appropriate content level to clarify the hybrid synergistic impact of CFs and NS. Therefore, combined with the Chinese code (DB22/T 5017-2019) and existing research achievements, the mixture proportions of RAC with incorporated CFs and NS are listed in Table 2.

### 2.3. Specimen Preparation

**Figure 2:**
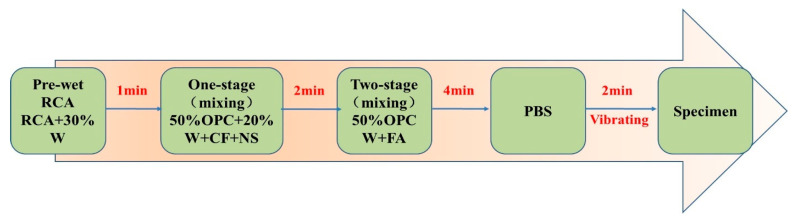
Schematic diagram of the mixing procedure for RAC.

To obtain RAC with uniformly dispersed NS, NS should be effectively dispersed before preparing CF-/NS-reinforced RAC. The weighed NS was mixed with water in a beaker, which was dispersed by an HN-500 ultrasonic nanomaterial disperser. The mixing procedure for preparing RAC was illustrated in Figure 2. The mixtures of RAC were poured into molds with a side length of 100 mm and compacted on a vibrating table. Subsequently, the RAC specimens were placed in a curing room with a relative humidity of >95% and a temperature of 20 ± 2 °C. The RAC specimens were demolded after 1 d and then cured in a standard curing environment (20 ± 2 °C and relative humidity of 98%) until the day of testing.

### 2.4. Testing Methods

#### 2.4.1. Mechanical Properties

The compressive strength and splitting tensile strength of RAC specimens were measured according to the Chinese standard GB/T50081-2019. After curing for 7, 28, and 90 d, at least three specimens for compressive and splitting tensile strength were tested with a TYA-2000 pressure testing machine, and the average value was obtained as the result with which to analyze the effect of CFs and NS on RAC.

#### 2.4.2. Microscopic Testing

The RAC samples for microstructural analyses were soaked in cold isopropanol to stop further hydration and then dried in an incubator at 50 ± 2 °C for 24 h before testing. The specimens for XRD and TG were mechanically ground into powder with a small size (≤200 μm). The phase composition of the samples was characterized with a Bruker D8 Advance instrument with scanning angles (2θ) ranging from 5° to 65° at a rate of 0.6 s/step. The thermogravimetry analysis of samples was carried out on an STA 449F5 thermogravimetry analyzer in a throughflow N_2_ atmosphere, and the temperature ranged from 30 °C to 900 °C at a heating rate of 10 °C/min. Samples with a size of about 3 mm in height and 5 mm in length and width were used to analyze the pore structure of RAC. An Auto Pore IV (9500) instrument was applied to determine the porosity and pore size distributions in RAC samples. The morphologies of RAC samples were observed via SEM on a Model Gemini 500.

The contents of Ca (OH)_2_ (W_P_) and chemically bound water (W_CBW_) were calculated with Equations (5)–(7), respectively:

(1) $$W_{P}=\frac{74}{18}\times \left(\frac{W_{380\;{}^{\circ}C}-W_{450\;{}^{\circ}C}}{W_{50{}^{\circ}C}}\right)\times 100\%$$

(2) $$W_{\mathrm{CBW}}=\frac{\frac{W_{50\;{}^{\circ}C}-W_{900\;{}^{\circ}C}}{W_{50\;{}^{\circ}C}}-{\mathrm{LOI}}_{\mathrm{BC}}}{1-{\mathrm{LOI}}_{\mathrm{BC}}}\times 100\%$$

(3) $${LOI}_{BC}=\left(1-\alpha \right){LOI}_{C}+\alpha {LOI}_{f}+\beta {LOI}_{NS}$$

where *LOI* represents the loss on ignition of raw materials. The *LOI_C_*, *LOI_f_*, and *LOI_NS_* are 0.015, 0.0495, and 0.0366, respectively. α and *β* are the substitution of fly ash and NS for cement.

## 3. Results and Discussion

### 3.1. Effect of CFs and NS on the Mechanical Properties of RAC

**Figure 3:**
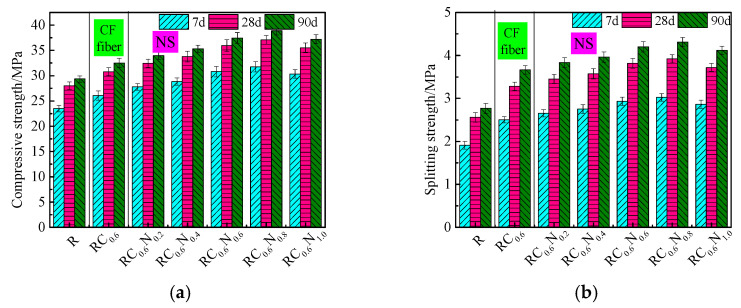
Compressive and splitting strength of RAC with CFs and NS. (**a**) Compressive strength; (**b**) splitting strength.

The effect of CFs/NS on the compressive strength and splitting tensile strength of the prepared RAC is shown in Figure 3. It was observed that the compressive strength of RC_0.6_ at 7, 28, and 90 d was 26.08 MPa, 30.77 MPa, and 32.51 MPa, which increased by 8.35%, 9.05%, and 9.32%, respectively, in comparison to R. Compared with R, the splitting tensile strength of RC_0.6_ increased by 21.12%, 22.36%, and 23.56%, respectively. The results were similar to those of [12], which showed that the addition of CFs significantly increased the static splitting tensile strength and dynamic splitting tensile strength, but that this was not obvious for the compressive strength. The results further demonstrated that the incorporation of CFs into RAC significantly impacted the splitting tensile strength. This may be due to the fact that (i) the CFs dispersed in RAC could form a network structure, which limited the generation and expansion of dry shrinkage cracks in the early hydrated process, leading to a reduction in the early defects of the RAC; (ii) the high elastic modulus of the CFs had a significant enhancement effect on the splitting tensile strength, which had a small enhancement effect on the compressive strength; and (iii) the friction between the CFs and RAC matrix could consume a certain amount of energy and then increase the toughness of the RAC according to the fiber spacing theory.

Compared with the single-doped CFs, the effect of the hybrid addition of the CFs and NS on the mechanical properties of RAC was more remarkable. Note that the compressive strength and splitting tensile strength of RC_0.6_N_0.8_ were the highest among all of the samples studied, which reached 37.08 MPa and 3.92 MPa at 28 d, increasing by 20.51% and 14.53%, respectively, in comparison to those of RC_0.6_. This was attributed to the “filling effect”, “nucleation effect”, and “chemical reaction” of NS. The particle sizes of NS were much smaller than those of cement, which filled the pores on the surface of the RAC and compacted the density of the ITZ. Meanwhile, the NS could provide more nucleation points at an early age, which accelerated cement hydration and facilitated the formation of C-S-H gel, resulting in improving the RAC compactness by filling the pores. In addition, NS could increase the volume fraction of high-density C–S–H gels (stacking density of 0.75) by facilitating cement hydration and linking low-density C–S–H gels [12] (stacking density of 0.64). The addition of 0.2 wt% NS increased the compressive strength and splitting tensile strength of the RAC by 3.93%~6.08% and 3.05%~5.21%, illustrating that the effect of a content of NS below 0.2 wt% was not obvious in terms of improving the compressive strength and splitting tensile strength of the RAC. When the content of NS increased from 0.8 wt% to 1 wt%, the compressive strength and splitting tensile strength of RC_0.6_N_1.0_ exhibited decreases of approximately 2.93%~4.43% and 2.53%~5.29% in comparison to those of RC_0.6_N_0.8_ at 7, 28, and 90 d, respectively. This was mainly because the small particle size and large specific surface area of NS would produce an agglomeration phenomenon when the content of NS was too high. NS agglomerates wrapped in water molecules would have inhibited the hydration of cement, which reduced the generation of hydration products and weakened the bond between the hydration products and RAC surfaces, leading to a reduction in the enhancement effect. Yao et al. [24] found that the addition of 0.6% NS increased the compressive strength, flexural strength, and splitting strength of coal gangue concrete at 7 d by 11.8%, 12.5%, and 13.4%, respectively. Fu et al. [17] previously reported that adding an appropriate amount of NS could improve the mechanical properties and durability performance of RAC by enhancing the ITZ compaction and optimizing the microstructure of the RAC. As can be seen from the above discussion, NS can function by modifying the mechanical properties of RAC, and the recommended dosage of NS is 0.8 wt%.

### 3.2. The Relationship Between the Compressive Strength and Splitting Tensile Strength of RAC

**Figure 4:**
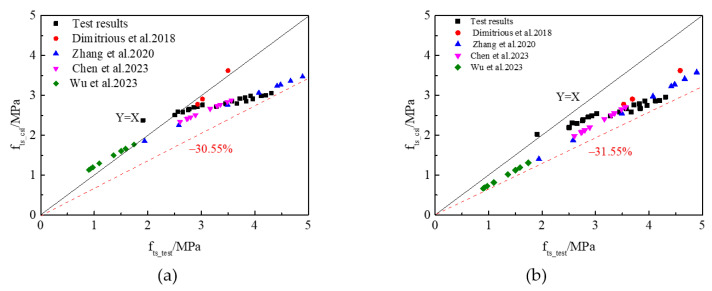
Comparison of experimental and predicted values. (**a**) ACI Committee318; (**b**) Chinese code.

Generally, the relationship between the splitting tensile strength and cube compressive strength is an important parameter in practical engineering applications. The ACI Committee code 318 for concrete structures proposes an equation, Equation (4), to predict the splitting tensile strength based on the corresponding cube compressive strength of normal concrete. The Chinese code proposed a similar equation, Equation (5), to calculate the relationship between the cube compressive strength and splitting tensile strength of normal concrete. The comparison between the theoretical results and test results of the splitting tensile strength of RAC calculated by Equations (4) and (5) is shown in Figure 4. It can be seen from Figure 4a,b that the formulas suggested by ACI Committee code 318 and the Chinese code had a poor prediction accuracy for RAC owing to the internal structure and failure mechanism of RAC generally differing from those of conventional concrete, resulting in the overestimation of its splitting tensile strength and then a reduction in the structural reliability. This being the case, the relationship between the splitting tensile strength and cube compressive strength of RAC should be revised. According to ACI Committee code 318 and the Chinese code, the relationship between the splitting tensile strength and compressive strength of RAC could be calculated by Equation (6):

(4) $$f_{ts}=0.19f_{c}^{0.75}$$

(5) $$f_{ts}=0.49f_{c}^{0.5}$$

(6) $$f_{ts}=\alpha f_{c}^{\beta }$$

where $f_{c}$ and $f_{ts}$ are the compressive strength and splitting tensile strength of concrete, respectively.

**Figure 5:**
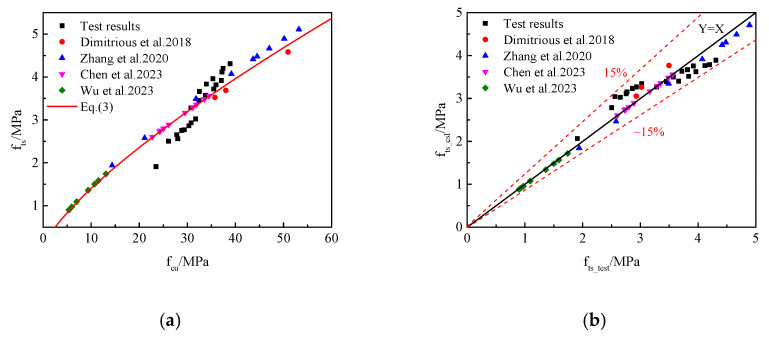
Relationship between the compressive strength and splitting strength of RAC. (**a**) Regression analysis; (**b**) comparison of the experimental and predicted values.

Equation (6) was used to fit the relationship between the splitting tensile strength and compressive strength of the experimental and available literature results of RAC samples (Figure 5a). Coefficients α and β and the correlation coefficient (R^2^) could be calculated via nonlinear regression analysis, and they were 0.25, 0.75, and 0.92, respectively. Thus, the relationship between the splitting tensile strength and compressive strength of RAC samples with CFs and NS could be displayed as in Equation (7):

(7) $$f_{ts}=0.25f_{c}^{0.75}$$

The comparisons between the prediction results calculated according to Equation (7) and the test results are shown in Figure 5b. As can be seen from Figure 5b, the ratio of the prediction results and test results of the splitting tensile strength of RAC presented a positive correlation, and the error was below ± 15%, which met the error requirements, indicating a good agreement between the experimental and prediction results. However, there were few investigations into studying the relationship between the splitting tensile strength and compressive strength of RAC with CFs and NS, and only limited data were available. Hopefully, Equation (7) can be checked and improved via the experimental results produced from later experimental studies.

### 3.3. Effect of CFs/NS on the Hydration Products of Cement

**Figure 6:**
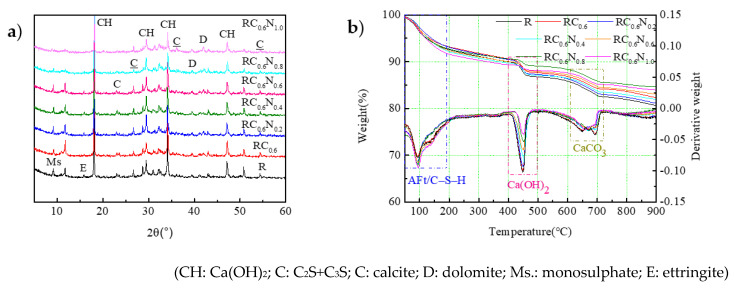
Effect of CFs/NS on the hydration products of cement. (**a**) XRD; (**b**) TG and derivative thermogravimetry (DTG).

The hydration product characteristics of cement directly determined the mechanical properties of RAC. In order to investigate the impact of CFs and NS on the hydration products of cement, the hydration products of RAC pastes cured for 28 d were comparatively tested via XRD and TG. As illustrated in Figure 6a, the XRD patterns showed calcium hydroxide (Ca(OH)_2_, CH), ettringite (AFt, E), monosulfatealuminate (Ms), and calcite (CaCO_3_). Some unhydrated C_3_S and C_2_S phases were also identified near the 2*θ* value of 32°–33° in the XRD patterns. The diffraction patterns of C-(A)-S-H gels did not appear in the XRD pattern due to the X-ray diffraction tests not being able to characterize the gel-like substances of the C-(A)-S-H gels. The results indicated that the incorporation of CFs had little effect on the hydration products. This was mainly attributed to the fact that the CFs were hydrophobic and hardly participated in the hydration reaction of cement. The diffraction patterns of CH, C_2_S, and C_3_S gradually decreased with the addition of NS, and the decrease was greater when the content of NS increased. The results indicated that NS was favored to facilitate the dissolution of the active amorphous phases and formation of the C-(A)-S-H gels. Consequently, the inclusion of NS mainly led to an acceleration in the hydration reaction of cement, attributed to the fact that (i) the pozzolanic reaction of CH and NS could promote the dissolution of the active amorphous phases of C_3_S and C_2_S, facilitating the formation of more C-(A)-S-H gels; (ii) the active phases of NS could create more latent reaction opportunities in the hydration of cement to form high-density C–S–H gel and Ms via a pozzolanic reaction and chemical promotion to consume C_3_A at the early stages; and (iii) the C-S-H gels can be bonded to each other and then form a network structure, which could be used as a diffusion and support medium for crystal growth, resulting in the accelerated dissolution of anhydrous phases and more pronounced expression as well as a higher generation rate of the C-S-H gel (including the high-density C–S–H gel), with a significant contribution to the mechanical properties of the RAC.

In order to quantitatively analyze the effect of the CFs and NS on the thermal decompositions of cement-based hydration products, the TG and derivative thermogravimetric (DTG) curves of RAC paste at 28 d were tested and are displayed in Figure 6b. It was clear that there were three apparent heat absorption peaks at 50~200 °C, 400~500 °C, and 600~750 °C, which corresponded to (I) the evaporation of water molecules and dehydration of Aft or C-S-H gel, (II) the decomposition of CH, and (III) the decomposition of produced and residual CaCO_3_, respectively. From the changing trends, the addition of CF had little impact on the mass loss at stages I, II, and III, implying that the CFs did not participate in the hydration reaction of cement. Compared with RC_0.6_, the RAC pastes containing different NS contents had a relatively higher mass loss at stage I, significantly decreased mass loss at stage II, and a small difference in the mass loss at stage III. Additionally, the increasing content of NS contributed an obvious impact on the mass loss at stages I and II of the RAC pastes. These results illustrated that the inclusion of NS could promote more occurrences of hydration reactions, resulting in the formation of more C-(A)-S-H gels and less CH.

**Figure 7:**
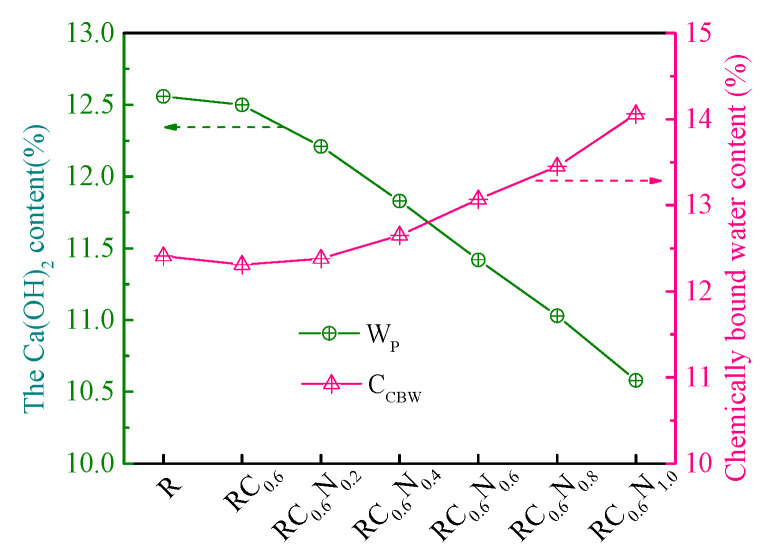
The content of Ca (OH)_2_ and chemically bound water in RAC.

As is commonly known, the contents of Ca (OH)_2_ (W_P_) and chemically bound water (W_CBW_) are parameters for evaluating the amount of hydration products, which were calculated by Equations (1)–(3). To further investigate the effect of CFs/NS on the amount of hydration products, the contents of Ca (OH)_2_ and chemically bound water were calculated and are shown in Figure 7. As displayed in Figure 7, it can be observed that the Ca (OH)_2_ and chemically bound water content of RC_0.6_ were 99.52% and 99.19%, respectively, of those of R, illustrating that the inclusion of CFs had little influence on the Ca (OH)_2_ and chemically bound water content, which is consistent with the XRD results. The chemically bound water content increased as the NS content increased, whereas the content of Ca (OH)_2_ decreased with more NS. For example, the chemically bound water content in RC_0.6_N_0.2_, RC_0.6_N_0.4_, RC_0.6_N_0.6_, RC_0.6_N_0.8_, and RC_0.6_N_1.0_ increased by 1.12%, 2.76%, 6.17%, 9.26%, and 14.21%, respectively, and the Ca (OH)_2_ content in RC_0.6_N_0.2_, RC_0.6_N_0.4_, RC_0.6_N_0.6_, RC_0.6_N_0.8_, and RC_0.6_N_1.0_ decreased by 2.32%, 5.36%, 8.64%, 11.26%, and 15.36%, respectively, compared to that of RC_0.6_, which further demonstrated that the addition of NS could facilitate the dissolution of anhydrous phases and the formation of C-(A)-S-H gels. The TG-DTG results verified that the addition of NS could effectively improve the hydration reaction of cement and that 0.8 wt% NS was the best choice for the CF-reinforced RAC, which was consistent with the mechanical property results.

### 3.4. Effect of CFs/NS on Pore Structures of RAC Samples

**Figure 8:**
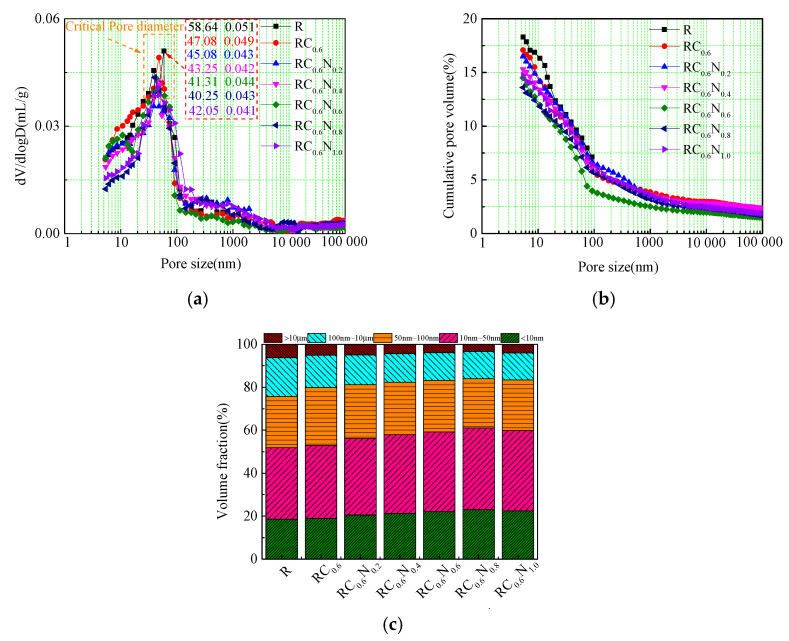
Effect of CFs/NS on the pore size distribution, pore proportion distribution, and porosity of cement paste. (**a**) The pore size distribution; (**b**) the cumulative pore volume; and (**c**) the pore proportion distribution.

Figure 8 displays the pore size distribution curves, cumulative porosity variation curves, and pore proportion distribution curves of the CF-/NS-reinforced RAC. The critical pore diameter was an important parameter for evaluating the concrete pore characteristics, which corresponded to the highest mercury intrusion rate per pore diameter change. As illustrated in Figure 8, the critical pore diameter and cumulative porosity of RC_0.6_ were 47.08 nm and 17.39%, respectively, close to those of R (48.06 nm and 18.04%). The results indicated that the addition of CFs had an insignificant optimization effect on decreasing the critical pore diameter and cumulative porosity. However, the compressive strength and splitting tensile strength of RC_0.6_ were obviously higher than those of RAC, which was not in accordance with the law that the lower porosity and the critical pore diameter of ordinary concrete correspond to high mechanical properties. This was mainly attributed to the fact that the crack inhibition action of the CFs inhibited the generation of micro-bubbles to a certain extent during the hardening process and enhanced the mechanical properties. When the NS and CFs were both added, the NS’s reducing effect on the critical pore diameter and porosity was stronger than the CF optimization effect on the critical pore diameter and porosity. It is worth noting that the critical pore diameter and porosity of RC_0.6_N_0.8_ were 12.98% and 40.25 nm, respectively, which decreased by 25.35% and 14.51%, respectively, compared to those of RC_0.6_. The critical pore diameter and the porosity of RC_0.6_N_1.0_ were 14.74% and 42.05 nm, respectively, implying that 1.0 wt% NS slightly decreased the optimization effect on the RAC pore structure. This result was consistent with the mechanical properties of RAC, which was because excessive NC would result in an agglomeration phenomenon and cause the insufficient hydration of cement.

To further quantitatively analyze the impact of CFs/NS on the pore distribution of the RAC, the pores present in the cementitious materials were divided into five groups according to previous studies, which were gel pores (0–10 nm), harmless capillary pores (10–50 nm), harmful capillary pores (50–100 nm), harmful pores (100 nm–10 µm), and more harmful pores (>10 µm). The percentage of gel pores, harmless capillary pores, and harmful capillary pores in RC_0.6_ was 18.98%, 33.89%, 26.98%, respectively, close to those of R (18.58%, 33.29%, and 23.84%), demonstrating that the incorporation of CFs did not participate in the hydration reaction and had little effect on the optimization of pores smaller than 100 nm in RC_0.6_. However, the percentages of harmful pores and more harmful pores in RC_0.6_ was 15.01% and 5.11%, and were increased by 16.65% and decreased by 13.17%, respectively, in comparison to those of R. The results showed that the addition of CFs could inhibit the generation of micro-bubbles to a certain extent during the hardening process, which was in agreement with the results of Niu et al. [25] and Xu et al. [26]. Compared to RC_0.6_, the RAC with CFs and NS had a better optimization effect on pores smaller than 50 nm, which was caused by the transformation of harmful capillary pores and harmful pores into harmless capillary pores and gel pores due to the “filling effect”, “nucleation effect”, and “pozzolanic reaction” of NS. The percentage of pores smaller than 50 nm in RC_0.6_N_0.2_, RC_0.6_N_0.4_, RC_0.6_N_0.6_, RC_0.6_N_0.8_, and RC_0.6_N_1.0_ increased by 6.32%, 9.56%, 11.96%, 15.65%, and 13.42%, respectively, compared to that of RC_0.6_. The results indicated that the addition of NS had a significant optimization effect on pores smaller than 50 nm, which was consistent with the conclusion illustrated by Chen et al. [27], that NS could effectively increase the percentage of pores smaller than 50 nm and decrease the porosity of cementitious materials.

### 3.5. Microstructure Analysis

**Figure 9:**
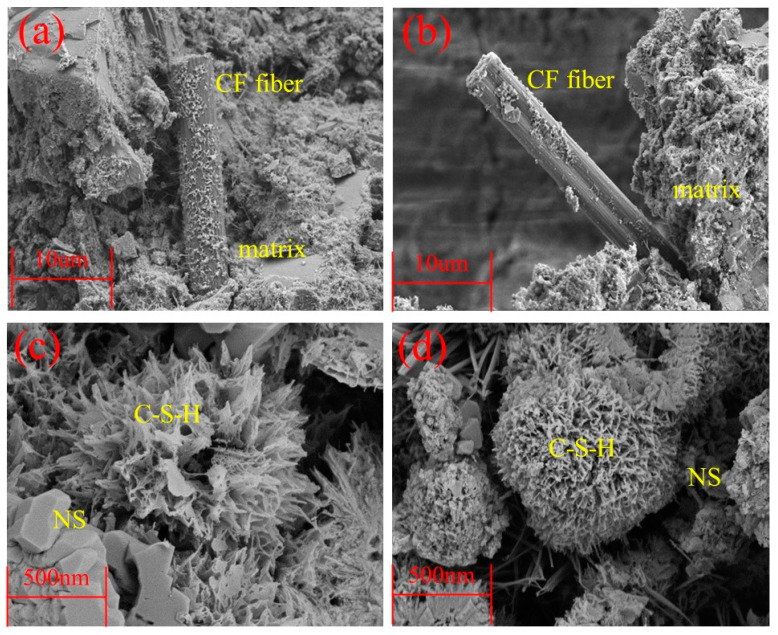
The morphologies of hydration influenced by CFs and NS. (**a**) The CFs and matrix; (**b**) the adhered hydration products on CFs; (**c**) the pores filled by NS and (**d**) the high-density C–S–H gels.

To investigate the effect of CFs/NS on the microstructure characteristics of RAC, the RAC samples were examined via scanning electron microscopy and are displayed in Figure 9. As displayed in Figure 9a, it can still be clearly observed that the CFs could bridge the microscale pores and cracks in the RAC matrix and restrain the further development of these defects during the hardening process, which was in accordance with the analysis in Section 3.1. Meanwhile, it can be found that the interfacial regions between the CFs and RAC were presented as being dense and the hydration products were tightly adhered to the CF surface, as shown in Figure 9b. The results implied that the CFs could form a three-dimensional network structure and strengthen the bonding strengths of the interfacial regions, which was in agreement with previous studies showing that an appropriate amount of CFs could form a thick network skeleton in RAC and restrict the transverse deformation of the RAC under compression [28,29,30,31,32,33]. As displayed in Figure 9c, it can be observed that NS filled the pores in the RAC matrix, which not only reduced the porosity of the RAC but compacted the surface of the RAC. Additionally, NS could link low-density C–S–H gels [12] (stacking density of 0.64) to form high-density C–S–H gels by functioning as linking units, as shown in Figure 9d. Lastly, the C-S-H gels (high-density C–S–H gels and low-density C–S–H gels) were strongly adhered on the CFs, which further improved the mechanical properties of the RAC [34,35]. Owing to the aforementioned functions of CFs and NS, the microscopic characteristics and macroscopic mechanical properties of the RAC can be obviously enhanced, and the enhancement mechanisms are summarized in Section 3.6.

### 3.6. Action Mechanism of CFs and NS

**Figure 10:**
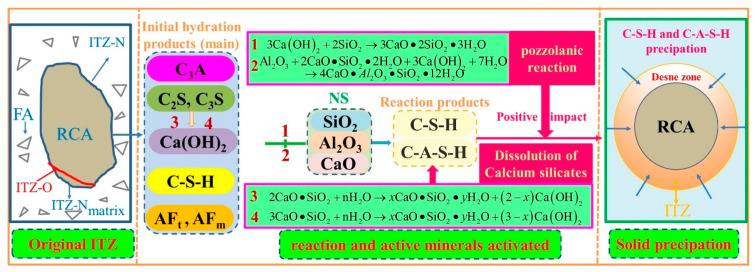
Reaction processes of NS enhancing the ITZ.

**Figure 11:**
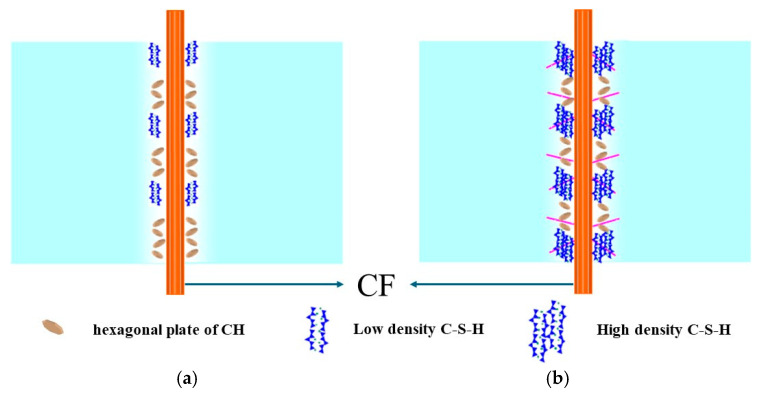
Effect of NS on the interface between CFs and the cement matrix. (**a**) CF surface with low-density C-S-H; (**b**) CF surface with high-density C-S-H.

As in the analysis mentioned in Section 3.5, the inhibitory effect on the initiation and expansion of cracks significantly increased when the CFs and NS were both added. The enhancement effect of NS on the RCA not only strengthened the bonding between the matrix and RCA but also enhanced the bonding between the matrix and CFs. This was attributed to the following: (i) NS could not only fill the pores in the ITZ but also easily decreased the migration of relevant ions to the RCA surface, resulting in the “filling effect” and “side wall effect” [36,37,38], which provided numerous nucleation sites and then increased the formation of more products on the RCA surface. (ii) The enhancement effect of NS on the RAC mainly involved four key steps, as illustrated in Figure 10. The pozzolanic reaction between NS, CH, and Al_2_O_3_ (see Equations (1) and (2) in Figure 10) could facilitate the formation of C-S-H gel and C-A-S-H gel, which turned capillary pores into gel pores to a significant extent. The proportions of unhydrated C_2_S and C_3_S in the cement were increased via the consumption of CH in pozzolanic reactions, which further promoted the generation of more C-S-H gel and improved the entire hydration degree of the cement. More importantly, NS could link low-density C-S-H gels [12] (stacking density of 0.64) to form high-density C-S-H gels by functioning as linking units. Finally, the thin hexagonal plate-like CH crystals could not provide effective mechanical strength for the RAC due to CH crystals being prone to fracture and easily forming microholes under loads. Consequently, the mechanical strength of the RAC was enhanced by the consumption of CH in the pozzolanic reactions of NS. (iii) NS enhanced the interface between the RAC matrix and CFs, which was more conducive to the effect of the CFs on crack suppression and further improved the strain rate effect under loads, as shown in Figure 11. Therefore, when the CFs and NS were both added, they not only inhibited the initiation and expansion of cracks in the RAC matrix but also significantly increased the resistance to crack expansion and the curvature of the crack expansion path in RAC, resulting in a decreased crack penetration ability and improved the mechanical properties. Owing to the aforementioned functions of CFs and NS, the microscopic characteristics and macroscopic mechanical properties of RAC could be obviously enhanced when appropriate levels of CFs and NS are both added.

## 4. Conclusions

1. The addition of the CFs and NS increased the mechanical properties of the RAC, and the improvement effect was most significant for the hybrid addition of the CFs and NS. The hybrid addition of 0.6 wt% CFs and 0.8 wt% NS increased the 28 d compressive strength and splitting strength of the RAC by 32.38% and 29.53%, respectively, and the optimum volume content of NS was 0.8 wt%.
2. The XRD and TG-DTG results confirmed that the addition of CFs had little effect on the hydration products of the cement. The chemically bound water content increased by 9.26% and the Ca(OH)_2_ content decreased by 11.26% when 0.8 wt% NS was added, which demonstrated that the addition of NS could facilitate the dissolution of anhydrous phases and the formation of C-(A)-S-H gels.
3. The critical pore diameter and cumulative porosity of RC0.6 were 47.08 nm and 17.39%, respectively, close to those of R, indicating that the addition of CFs had little effect on decreasing the porosity of the RAC. In contrast, the critical pore diameter and porosity of RC0.6N0.8 were 12.98% and 40.25 nm, which decreased by 25.35% and 14.51%, respectively, compared to those of RC0.6, demonstrating that the incorporation of NS significantly optimized the pore structure of the RAC.
4. When the CFs and NS were both mixed, NS could improve the bonding between the RAC matrix and CFs, which was more conducive to enhancing the mechanical properties of the RAC and could effectively contribute to the reuse of construction waste in the concrete industry.

The results of this study indicated that it was technically viable to improve the mechanical properties and microstructure of RAC by incorporating CFs and NS. The incorporation of hybrid CFs and NS could improve the mechanical strength of RAC and optimize its microstructure, in addition to promoting the utilization of RAC in the concrete industry, which has significant economic and social benefits. For future work, we propose that more attention is paid to improving the durability performance of RAC.

## Figures and Tables

**Table 1:** Chemical compositions and physical properties of cement and fly ash.

Composition	SiO_2_	Al_2_O_3_	Fe_2_O_3_	CaO	MgO	SO_3_	SiO_2_	LOI	Compressive Strength/MPa		Flexural Strength/MPa	
Cement	18.80	5.15	3.345	57.83	0.916	3.95	18.80	3.95	3d	28d	3d	28d
Fly ash	50.96	29.38	4.21	6.58	1.13	0.481	50.96	2.79	26.5	45.3	5.6	7.8

**Table 2:** Mix proportions of RAC (kg/m^3^).

Mixture	C	FA	W	S	CA	PBS	CF	NS
R	380	120	237	720	900	3.8	0	0
RC_0.6_							2.28 (0.6 wt%)	0
RC_0.6_N_0.2_								0.76 (0.2 wt%)
RC_0.6_N_0.4_								1.52 (0.4 wt%)
RC_0.6_N_0.6_								2.28 (0.6 wt%)
RC_0.6_N_0.8_								3.04 (0.8 wt%)
RC_0.6_N_1.0_								3.8 (1.0 wt%)

## Data Availability

The original contributions presented in the study are included in the article; further inquiries can be directed to the corresponding author.
